# Alpha-Lipoic Acid Downregulates IL-1β and IL-6 by DNA Hypermethylation in SK-N-BE Neuroblastoma Cells

**DOI:** 10.3390/antiox6040074

**Published:** 2017-09-26

**Authors:** Simona Dinicola, Sara Proietti, Alessandra Cucina, Mariano Bizzarri, Andrea Fuso

**Affiliations:** 1 Department of Experimental Medicine, Sapienza University of Rome, Viale Regina Elena 324, 00161 Rome, Italy; simona.dinicola@uniroma1.it (S.D.); mariano.bizzarri@uniroma1.it (M.B.); 2 Department of Surgery “Pietro Valdoni”, Sapienza University of Rome, Via Antonio Scarpa 16, 00161 Rome, Italy; sara.proietti@uniroma1.it (S.P.); alessandra.cucina@uniroma1.it (A.C.)

**Keywords:** DNA methylation, IL-1b, IL-6, epigenetics, inflammation, lipoic acid, neuroblastoma

## Abstract

Alpha-lipoic acid (ALA) is a pleiotropic molecule with antioxidant and anti-inflammatory properties, of which the effects are exerted through the modulation of NF-kB. This nuclear factor, in fact, modulates different inflammatory cytokines, including IL-1b and IL-6, in different tissues and cell types. We recently showed that IL-1b and IL-6 DNA methylation is modulated in the brain of Alzheimer’s disease patients, and that IL-1b expression is associated to DNA methylation in the brain of patients with tuberous sclerosis complex. These results prompted us to ask whether ALA-induced repression of IL-1b and IL-6 was dependent on DNA methylation. Therefore, we profiled DNA methylation in the 5’-flanking region of the two aforementioned genes in SK-N-BE human neuroblastoma cells cultured in presence of ALA 0.5 mM. Our experimental data pointed out that the two promoters are hypermethylated in cells supplemented with ALA, both at CpG and non-CpG sites. Moreover, the observed hypermethylation is associated with decreased mRNA expression and decreased cytokine release. These results reinforce previous findings indicating that IL-1b and IL-6 undergo DNA methylation-dependent modulation in neural models and pave the road to study the epigenetic mechanisms triggered by ALA.

## 1. Introduction

The role of alpha-lipoic acid (ALA), also known as thioctic acid, as an antioxidant and anti-inflammatory molecule is largely known [1]. In particular, it is an object of great interest for its high antioxidant capacity and peculiar ability to challenge the production of free radicals in both lipophilic and hydrophilic environments, and ability to maintain its antioxidant properties both in its oxidised and reduced form [2,3,4].

Antioxidant action of ALA is mediated by two essential nuclear factors: nuclear factor erythroid 2-related factor 2 (Nrf2) and nuclear factor kappa-light chain-enhancer of activated B cells (NF-kB) [5,6,7,8,9,10]. Amongst the many cytokines under transcriptional control of these NFs, IL-1β and IL-6 represent two relevant pro-inflammatory cytokines involved in pathological processes in many different tissues and cell types [11,12,13,14,15]. These two genes are of particular interest regarding our scopes, since it is already known that they are regulated by ALA and are involved in neurodegenerative processes [16]. Therefore, ALA has been proposed as useful in adjuvant treatment for the management of different human diseases [17], including diabetes, cardiovascular disease, bone loss, inflammatory chronic disease, and others, including neurodegenerative diseases and neuropathy [18,19]. Overall, the preliminary data provided the rationale for ALA-based anti-aging treatment [9,20].

The preading of neurodegenerative diseases associated with aging, particularly Alzheimer’s disease (AD), has become a public health priority and challenge. AD affects approximately 44 million people worldwide, with the rate estimated to increase, which could result in 115 million people being affected by 2050 [21]. AD is characterised by progressive cognitive decline, along with the presence of intracellular deposits of fibrillary hyper-fosforylated Tau and extracellular deposits of aggregated amyloid-β (Aβ) protein [22] in the brains of infected subjects. No effective therapies are known as of yet. Due to the lack of early diagnostic biomarkers and predictive specific symptoms, AD can remain asymptomatic for a long time before cognitive decline is evident. Therefore, early intervention and preventive strategies could help to counteract the molecular processes at the origin of this pathology [23]. Many different molecular pathways are dysregulated in AD, and could be potentially relevant in planning intervention aimed at preventing and/or curing the disease. Oxidative stress represents one of the major pathogenic mechanisms associated with AD pathological processes; although it is not yet completely clear how much oxidation can be causative or consequential of the disease [24,25]. From a causative perspective, it is known that oxidative stress is responsible for increased Aβ production and accumulation. However, Aβ can, in turn, increase oxidative stress per se [26,27]. Therefore, several antioxidant molecules are currently under scrutiny for their possible beneficial effects in the treatment of AD and neurodegenerative diseases.

Oxidative stress is generally associated with inflammation in AD and neurodegeneration, since both pro- and anti-inflammatory cytokines are modulated by oxidative stress and antioxidant response [28]. It is noteworthy that IL-1β and IL-6 are amongst the cytokines modulated by the ALA and are also involved in the inflammatory processes associated with AD and neurodegeneration [29,30,31]. In particular, IL-1β and IL-6 showed characteristic expression patterns at mRNA and protein levels in the brain of AD patients at different stages of the pathology [27]. It was possible to demonstrate that the specific patterns of mRNA expression correlated with the DNA methylation patterns in the promoters of the two genes [32]. This finding is indeed in agreement with the already known observation that IL-1β and IL-6 expression can be regulated by DNA methylation or other epigenetic mechanisms in different tissues and conditions [33,34,35,36,37]. 

Recently, we demonstrated that ALA-dependent regulation of IL-1β and IL-6 expression is associated to the modification of the DNA methylation of the respective promoters; specifically, we observed that ovarian epithelial cells treated with ALA undergo hypermethylation of IL-1β and IL-6 5’-flanking regions and consequent down-regulation at mRNA and protein levels [38]. On this basis, we aimed at demonstrating that the same epigenetic mechanism was associated with ALA-dependent down-regulation of IL-1β and IL-6 in neuronal cells. Therefore, we treated SK-N-BE human neuroblastoma cells, previously used as a model to study AD-associated mechanisms with ALA [39,40,41]. We investigated the DNA methylation profile by bisulphite transformation, and the expression of IL-1β and IL-6 at mRNA and protein levels by real-time polymerase chain reaction (RT-PCR) and Enzyme-Linked Immunosorbetn Assay (ELISA), respectively.

## 2. Materials and Methods 

### 2.1. Cell Cultures

According to the experimental plan, human neuroblastoma SK-N-BE cells (acquired from the American Type Culture Collection, Manassas, VA, USA) were plated in 10% Foetal Calf Serum (FCS)-containing medium, and after 24 h of growth, the cells were shifted to control medium (10% FCS, 1/1000 dimethylsulphoxyde, DMSO) or ALA 0.5 mM (dissolved in DMSO) supplemented medium. After 24 h of culture, cells were collected and stored at −80 °C for the following DNA or RNA purification. At 24 h, supernatants were also collected and stored at −80 °C for the following ELISA test. For each assay, three samples per condition were assessed, and each experiment was independently repeated three times. ALA was supplied by Lo.Li.Pharma (Rome, Italy) in a racemic mixture (*R*/*S*, 1:1) and dissolved in DMSO at a concentration of 500 mM (ALA solubility in DMSO: 1.5 M).

### 2.2. DNA Methylation Study by Bisulphite Modification and Genomic Sequencing

DNA isolation from cells was performed using the DNeasy Blood and Tissue Kit (Cat. #69504, Lot #136269927, Qiagen, Milan, Italy) and the Qiacube instrument (Qiagen, Milan, Italy). 

Bisulphite analysis of IL-1B and IL-6 promoter methylation was performed as previously described [26,37] using the EpiTect Bisulphite kit; PCR products obtained after bisulphite conversion were cloned using the PCR Plus Cloning Kit (both from Qiagen, Milan, Italy). At least ten positive bacterial clones per experimental condition were sequenced using M13 primers (in service by PRIMM, Milan, Italy).

The methylation patterns were assessed by recognition of the modified cytosine residues by comparing the sequencing outputs with Genbank DNA sequences; for each clone, methylation was reported as 1/0 value in an Excel spreadsheet (methylated: 1; unmethylated: 0). Methylation percentage was calculated for each sample at single cytosine level by the formula: (n° methylC/n° sequenced clones) × 100. Finally, we calculated the average methylation percentage over the experimental repetition for each experimental group. 

Methylation insensitive primers (MIPs) used for bisulphite analysis were already described [26,37]; these primers were designed to assess the methylation status of the plus (5′ → 3′) DNA strand. Positive and negative controls necessary to guarantee complete and efficient bisulphite conversion were prepared as previously described [26,37], and were treated and analysed in parallel with the experimental samples.

### 2.3. mRNA Expression Study by Real-Time PCR

RNA isolation from cells was performed using the RNeasy mini kit (Qiagen, Milano, Italy) and cDNA synthesised as previously described; 1 μg of total cDNA was used for real-time reactions; analyses were performed in triplicate for each sample as previously described [37]. cDNA levels were normalised to the β-actin control, and presented as the fold increase (ratio of the experimental gene value/β-actin gene value) over the control sample. GAPDH and 18S were also used to normalise the PCR reactions with comparable results (Supplementary Figure S1).

### 2.4. Cytokines Production Study by ELISA Test

Levels of cytokines released in the culture medium were determined by ELISA following the manufacturer’s instructions, using the following kits: IL-1β beta, sensitivity: 6.5 pg/mL (Abcam, ab46052); IL-6, sensitivity: 0.7 pg/mL (Quantikine, R&D, D6050). ELISA plates were analysed by a microtiter plate reader (Opsys MR; Dinex Technologies, Chantilly, VA, USA). 

### 2.5. Statistical Analysis

Statview 5.0 (SAS Institute, Milan, Italy) statistical software was used to apply the statistical analysis as previously described [31,38]. Briefly, methylation data were analysed by Mann–Whitney non-parametric tests; One-way Analysis of Variance (ANOVA) and Tuckey’s post-test were applied to calculate significant differences in real-time PCR and in ELISA assays; Pearson’s correlation was computed to assess the correlation between DNA methylation and mRNA expression. 

## 3. Results

**Figure 1:**
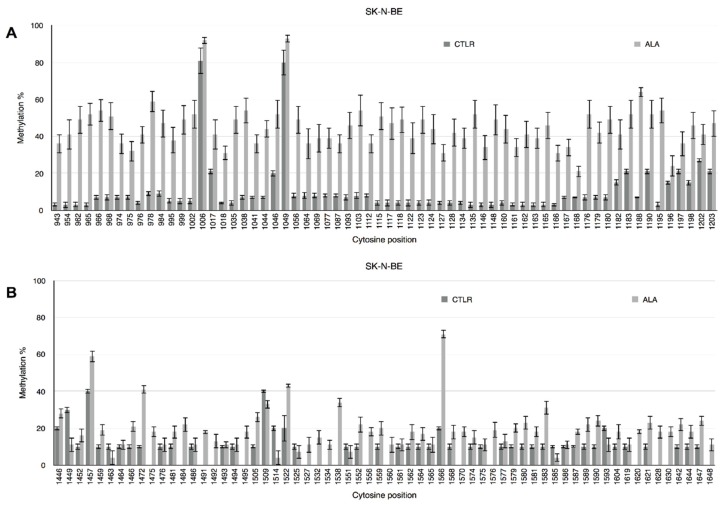
Methylation pattern of *IL-1β* and *IL-6* 5′-flanking regions. CpG and non-CpG site-specific methylation pattern expressed as percent methylation for each cytosine in the investigated region of the human *IL-1β* (**A**) and *IL-6* (**B**) promoters. Cytosine position in the reference sequence is indicated below the x-axis. Dark grey columns represent control cells, light grey columns represent alpha-lipoic acid (ALA)-treated cells.

DNA methylation status of CpG and non-CpG moieties in the 5′-flanking regions of the *IL-1β* and *IL-6* genes has been studied by bisulphite treatment of genomic DNA purified from human neuroblastoma SK-N-BE cells, cultured for 24 h in control or 0.5 mM ALA supplemented medium. Methylation profiles at single cytosine level are reported in Figure 1. Although both gene promoters show significant hypomethylation associated to ALA treatment, the difference in IL-1β (Figure 1A) methylation profile in ALA vs. control is more marked than in IL-6 (Figure 1B) promoter. In fact, Mann–Whitney test reports higher statistical significance (U = 9.00, *p* < 0.001) for IL-1β than for IL-6 (U = 9.00, *p* < 0.05). As in previous experiments from our laboratory [26,37], differences in methylation levels were observed even at nonCpG sites, that actually also show the greater differences with respect to the CpG moieties. 

**Figure 2:**
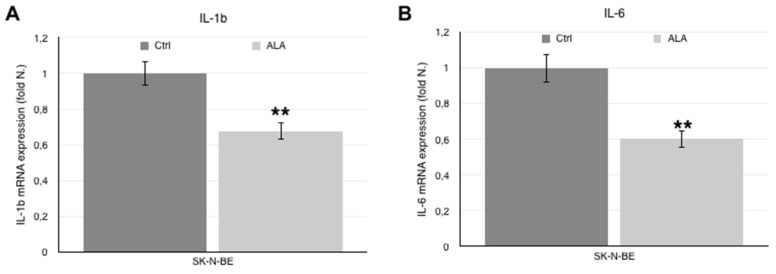

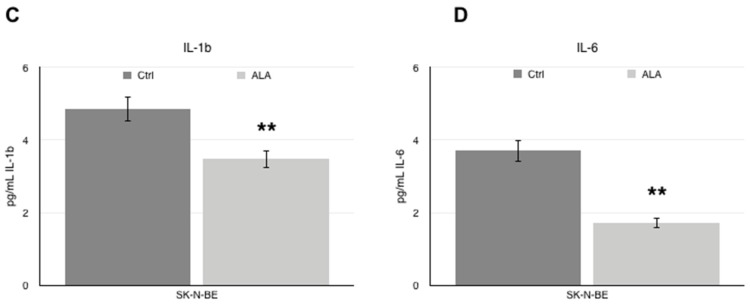
mRNA expression and protein levels of IL-1β and IL-6. *IL-1β* (**A**) and *IL-6* (**B**) mRNA expression levels, as determined by real time-PCR, in SK-N-BE cells treated with control (dark grey columns) and ALA supplemented (light grey columns) medium. IL-1β (**C**) and IL-6 (**D**) protein levels in the culture medium, as determined by ELISA test, SK-N-BE cells treated with control (dark grey columns) and ALA supplemented (light grey columns) medium. Histograms indicate the mean value ± SEM. ** *p* < 0.001 vs. Ctrl.

As shown in Figure 2, *IL-1β* (Figure 2A) and *IL-6* (Figure 2B) gene expression is significantly down-regulated in ALA-treated cells and is therefore inversely correlated to DNA methylation (*IL-1β: r* = 0.96; *IL-6: r* = 0.81, *p* < 0.01).

The levels of the two cytokines secreted in the culture medium was assessed by ELISA tests and is concordant with mRNA levels; indeed, IL-1β (Figure 2C) and IL-6 (Figure 2D) are more abundant in control medium and lower in the medium of cells treated with 0.5 mM ALA.

## 4. Discussion

The data herein reported adds to an increasing amount of literature demonstrating that the expression of IL-1β and IL-6 is modulated by epigenetic mechanisms. Specifically, we showed that treating SK-N-BE cells with ALA induces hypermethylation of IL-1β and IL-6 5′-flanking regions, and that hypermethylation is associated with reduced mRNA expression and protein release in the culture medium. Taking advantage of a modified bisulphite assay [42], we also confirmed that in human neuroblastoma cells, the observed modulation of the DNA methylation patterns does not only affect the cytosines associated with the CpG dinucleotides. Discrete and dynamically changing non-CpG methylation was indeed observed in both promoter regions, as previously shown in different experimental models for the same genes [26,37,38] and for other genes [42,43].

Non-CpG methylation has been, so far, considered as restricted to embryonic tissues and stem cells [44,45,46]. More recently, growing evidence indicates that unexpectedly high levels of non-CpG methylation (25–35% of total DNA methylation) are present in the adult brains of both mice and humans [47,48]. At present, we can affirm that non-CpG methylation plays a functional role also in adult mammalian tissues [49,50], including human brain [51].

The observation that IL-1β and IL-6, up-regulated in the inflammation response occurring during neurodegenerative processes, undergo epigenetic regulation through modulation of the DNA methylation pattern reinforces the idea that DNA methylation plays a central role in the pathological processes responsible for the onset of neurodegenerative diseases [52,53,54,55]. 

ALA was already indicated as a potential therapeutic agent in aging-associated neurodegenerative disorders [18,19,20], and it was successfully studied in neuroblastoma cell models, showing beneficial effects against oxidative stress, improving the antioxidant response of the cells [56,57,58,59]. Here, we disclosed a further biomolecular mechanism responsible for the anti-oxidant and anti-inflammatory effects of ALA. ALA concentration used in the present work (0.5 mM) can be considered quite high, although a large range of doses can be found described in literature, ranging from few micromolar to even 4 mM; the concentration of the active (*R*)-(+)-enantiomer in our experiment is expected to be 0.25 mM. Preliminary (data not shown) experiments in different cell cultures showed that ALA concentration ranging from 0.1 to 4 mM had no side effects on cell viability and growth.

The use of cancer cell lines represents a major limit, although it is also a necessary step in order to be able to perform ex vivo and in vivo studies. A second limitation of the present work is represented by the fact that no stress inducers were used to exacerbate inflammation in cell cultures, although cancer cells already show high inflammation levels with respect to normal cells. This seems particularly puzzling, since IL-1β secretion is known to be under regulation of different specific mechanisms triggered by inflammatory stimuli, including canonical and non-canonical pathways; whether the observed secretion in basal conditions is a consequence of the cancerous nature of the model used, remains to be ascertained. However, the aim of the present work was to disclose the existence of the epigenetic mechanisms associated to ALA supplementation. Further studies are obviously necessary to translate the observations and the mechanisms reported here, firstly in pre-clinical in vivo models of neurodegenerative diseases, and then in humans; nevertheless, this data strongly points out the potential of ALA as an elective antioxidant to be used to complement the therapies currently in use for the treatment of neurodegenerative diseases and in the preventive treatment of aging.

## Supplementary Materials

The following are available online at www.mdpi.com/2076-3921/6/4/74/s1, Figure S1: mRNA expression levels, as determined by Real Time-PCR, in SK-N-BE cells treated with control (dark grey columns) and ALA supplemented (light grey columns) medium using GAPDH (**A**,**B**) and 18S (**C**,**D**) for normalization. Histograms indicate the mean value ± s.e.m. ** *p* < 0.001 vs. Ctrl.
