# Supplementary material for: Alpha-Lipoic Acid Downregulates IL-1β and IL-6 by DNA Hypermethylation in SK-N-BE Neuroblastoma Cells
Source: Antioxidants (Basel). 2017 Sep 26;6(4):74. doi: 10.3390/antiox6040074 (PMC5745484; doi:10.3390/antiox6040074)
Supplement: Supplementary file 1 [file antioxidants-06-00074-s001.pdf]

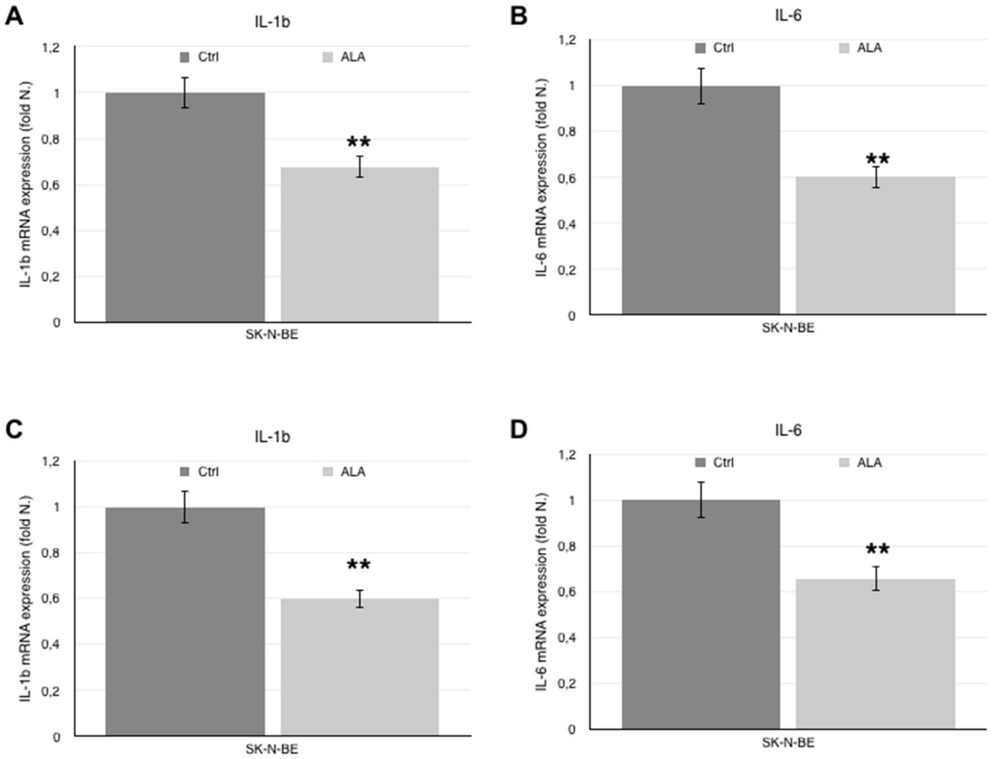

Supplementary Figure 1: mRNA expression levels, as determined by Real Time-PCR, in SK-N-BE cells treated with control (dark grey columns) and ALA supplemented (light grey columns) medium using GAPDH (A, B) and 18S (C, D) for normalization. Histograms indicate the mean value ± s.e.m. **: $p<0.001$ vs. Ctrl.
